# Heterologous *in vitro* fertilization and embryo production for assessment of jaguar (*Panthera onca* Linnaeus, 1758) frozen-thawed semen in different extenders

**DOI:** 10.1590/1984-3143-AR2021-0093

**Published:** 2022-03-21

**Authors:** Maria Valéria de Oliveira Santos, Herlon Victor Rodrigues Silva, Luana Grasiele Pereira Bezerra, Lhara Ricarliany Medeiros de Oliveira, Moacir Franco de Oliveira, Nilza Dutra Alves, Lúcia Daniel Machado da Silva, Alexandre Rodrigues Silva, Alexsandra Fernandes Pereira

**Affiliations:** 1 Laboratório de Biotecnologia Animal, Universidade Federal Rural do Semi-Árido – UFERSA, Mossoró, RN, Brasil.; 2 Laboratório de Reprodução de Carnívoros, Universidade Estadual do Ceará – UECE, Fortaleza, CE, Brasil.; 3 Laboratório de Conservação de Germoplasma Animal, Universidade Federal Rural do Semi-Árido – UFERSA, Mossoró, RN, Brasil

**Keywords:** big cat, biobanks, semen cryopreservation, *in vitro* interspecific embryo production

## Abstract

Heterologous *in vitro* fertilization (IVF) is an important tool for assessing fertility of endangered mammals such as the jaguar, considering difficult access to females for artificial insemination and to obtain homologous oocytes. We aimed to evaluate the fertility of jaguar sperm cryopreserved with different extenders, using domestic cat oocytes to assess the development of hybrid embryos. Semen from four captive jaguars was obtained by electroejaculation. Samples were cryopreserved in powdered coconut water (ACP-117c) or Tris extender containing 20% egg yolk and 6% glycerol. Thawed spermatozoa were resuspended (2.0 × 10^6^ spermatozoa/mL) in IVF medium and co-incubated with cat oocytes matured *in vitro* for 18 h. Presumptive zygotes were cultured for 7 days. After 48 h, cleavage rate was evaluated, and non-cleaved structures were stained for IVF evaluation. On days 5 and 7, the rate of morula and blastocyst formation was assessed. Data were analyzed using the Fisher exact test (*p* < 0.05). No difference was observed between ACP-117c and Tris extenders, respectively, for oocytes with 2nd polar body (2/51, 3.9 ± 2.9% *vs*. 2/56, 3.6 ± 3.1%), pronuclear structures (5/51, 9.8 ± 4.7% *vs*. 8/56, 14.3 ± 8.0%), and total IVF rates (7/36, 19.4 ± 5.0% *vs.* 10/37, 27.0 ± 13.8%). All the samples fertilized the oocytes, with 22.9 ± 3.2% (16/70) and 16.7 ± 3.6% (12/72) cleavage of mature oocytes for ACP-117c and Tris extenders, respectively. Morula rates of 4.3 ± 2.3% (3/70) and 5.6 ± 2.2% (4/72) were observed for ACP-117c and Tris, respectively. Only the Tris extender demonstrated blastocyst production (2/12, 16.7 ± 1.5% blastocyst/cleavage). We demonstrated that jaguar ejaculates cryopreserved using ACP-117c and Tris were suitable for IVF techniques, with blastocyst production by ejaculates cryopreserved in Tris. This is a first report of embryos produced *in vitro* using jaguar sperm and domestic cat oocytes through IVF.

## Introduction

The capacity to fertilize oocytes is one of the main indicators of sperm competence. Thus, *in vitro* fertilization (IVF) represents an important tool for predicting animal *in vitro* fertility (Pope et al., 2006). Nevertheless, for wild species, it is very difficult to obtain female gametes, mainly due to low availability of animals and lack of knowledge about the reproductive physiology of the female (Johnston et al., 1991). For this reason, heterologous IVF using oocytes of domestic species can be used for the development of semen-processing protocols in wild mammals (Stoops et al., 2007; Amstislavsky et al., 2018), such as the jaguar (*Panthera onca* Linnaeus, 1758).

The jaguar is considered the third largest felid in the world and plays an important ecological role in the ecosystems it inhabits (Gutiérrez-González and López-González, 2017). This carnivore is classified as a near-threatened species according to the International Union for Conservation of Nature Red List (Quigley et al., 2017). Moreover, in the Cerrado biome, the jaguar is classified as endangered, and in the Caatinga and Atlantic Forest biomes, it is critically endangered (Morato et al., 2013). Nevertheless, its population continues to decrease due to constant threat caused by habitat loss, forest fires and hunting for the illegal trade (Romero‐Muñoz et al., 2018; Barlow et al., 2020). Thus, strategies for conservation of this species in the view One Conservation concept are necessary, which consist of the interconnection among *ex-situ* and i*n-situ* plans, anthropic actions, and research in conservation areas (Pizzutto et al., 2021). Among the *ex-situ* strategies, the cryopreservation of semen is widely used for the applications of *in vivo* and *in vitro* reproductive biotechnologies (Araujo et al., 2020; Silva et al., 2020).

Recently, improvements have been reported in jaguar semen cryopreservation protocols (Araujo et al., 2020; Silva et al., 2020). Silva et al. (2020) observed that a tris-hydroxymethyl-aminomethane (Tris) extender allows better maintenance of the functionality of post-thawed sperm and improves the ability of sperm to bind to chicken egg perivitelline membrane compared to powdered coconut water (ACP-117c). Nevertheless, although these parameters are correlated with fertility, it is important to evaluate the capacity of jaguar spermatozoa to fertilize oocytes and initiate embryonic development.

To date, only few studies have reported the collection and maturation of jaguar oocytes (Johnston et al., 1991; Morato et al., 2002; Jorge-Neto, 2019). Therefore, heterologous IVF with domestic cat oocytes is a more accessible and safer alternative to assess the *in vitro* fertility of jaguar sperm. This methodology has already been used for several species of wild felids, such as the flat-headed cat (*Prionailurus planiceps*) (Thuwanut et al., 2011) and puma (*Puma concolor*) (Duque et al., 2018), in which hybrid embryos were able to develop to the blastocyst stage. On the other hand, for the bobcat (*Lynx rufus*) (Gañán et al., 2009) and Far‐Eastern wildcat (*Prionailurus bengalensis euptilurus*) (Amstislavsky, et al., 2018), only morulae were obtained after IVF with oocytes from domestic cats.

Thus, the aim of this study was to evaluate the *in vitro* fertility of jaguar semen cryopreserved using two different extenders (ACP-117c and Tris). We examined the fertilization competence of frozen-thawed sperm by studying its ability to fertilize oocytes of domestic cats and promote the development of hybrid embryos.

## Material and methods

Unless otherwise stated, the reagents used in this study were obtained from Sigma-Aldrich (St. Louis, USA).

### Animals

This study was approved by the Ethics Committee of the State University of Ceara (No. 5098414/2016) and by the Brazilian System of Authorization and Information of Biodiversity (No. 54741–1). Four male jaguars from zoos in different cities in north-eastern Brazil were used. One was from an Ecological Park – EcoPoint, Fortaleza, CE, Brazil (3° 43′ S, 38° 30′ W), weight: 66 kg, age: 17 years; one from the Teresina Zoobotanical Park, Teresina, PI, Brazil (5º 05′ S, 42º 48′ W), weight: 70 kg, age: 8 years; one from the Arruda Câmara Zoobotanical Park, João Pessoa, PB, Brazil (7º 06′ S, 34º 51′ W), weight: 69 kg, age: 9 years; and one from the Dois Irmãos Park, Recife, PE, Brazil (8º 03′ S, 34º 52′ W), weight: 68 kg, age: 4 years. Each animal was subjected to the usual management practices of each institution, with regular provision of red meat or slaughtered chicken as well as vitamin supplementation. Water was provided ad libitum.

### Anesthesia and semen collection

The animals were chemically restrained using blowpipe darts for intramuscular (IM) delivery of dexmedetomidine (Dexdomitor®, Zoetis, Campinas, Brazil) at a dose of 0.04 mg/kg, combined with ketamine hydrochloride (Ketalar®, Pfizer, São Paulo, Brazil) at a dose of 5 mg/kg. When necessary, one-third of the initial dose was administered to maintain anesthesia. To reverse the anesthesia after semen collection, yohimbine was administered at a dose of 0.4 mg/kg, IM (Lueders et al., 2012; Silva et al. 2019). Semen was collected according to the electroejaculation method described by Silva et al. (2019) using an electromechanical device (Autojac V2®, Neovet, Uberaba, Brazil). The semen from one collection of each animal was used for the experiment.

### Semen processing and cryopreservation

Immediately after collection, semen samples of each animal were evaluated for volume, color, concentration using Neubauer chamber, subjective motility by microscopy, and normal morphology using Bengal Rose (Silva et al., 2019). The entire process of dilution and freezing was performed as described by Silva et al. (2020). Briefly, the ejaculates were divided into two aliquots and diluted (1:1) either in ACP-117c (ACP Biotecnologia, Fortaleza, Brazil) or Tris (Sigma, St. Louis, USA). Samples were centrifuged at 300×*g* for 10 min to remove excessive seminal plasma and resuspended in the same extender (ACP-117c or Tris), with the addition of 20% egg yolk (1:1). The samples were stored in a thermal box and equilibrated for 40 min at 15 °C (cooling rate: -0.4 °C/min). Next, the samples were added to ACP-egg yolk or Tris-egg yolk plus glycerol (final concentration of 6%) and equilibrated for 30 min at 5 °C (cooling rate: -0.2 °C/min). Subsequently, the samples were packed into 0.25 mL plastic straws and placed horizontally, 5 cm above the surface of liquid nitrogen, in a Styrofoam box and maintained for 5 min. Finally, the straws were stored in liquid nitrogen.

Frozen straws were thawed by exposure to air for 10 s and then a 37 °C water bath for 30 s (Thiangtum et al., 2006). The sperm samples were dispensed in a microtube pre-warmed to 37 ºC and diluted (1:3) by slow addition (drop by drop) of Tyrode's albumin lactate pyruvate medium (TALP medium). The TALP medium contained 125 mM NaCl, 3.2 mM KCl, 2 mM NaHCO_3_, 0.4 mM NaH_2_PO_4_, 10 mM Na lactate, 2 mM CaCl_2_, 0.5 mM MgCl_2_, 10 mM HEPES, 0.6% bovine serum albumin (BSA), 0.2 mM sodium pyruvate, and 1% antibiotic–antimycotic solution (Sowińska et al., 2017). The sperm samples were centrifuged (500×*g* for 5 min), and the pellets thus obtained were resuspended in TALP medium. Total sperm motility was evaluated using a computer-assisted sperm analyser (CASA) (IVOS 7.4G; Hamilton-Thorne Research, Beverly, USA). The settings of the instrument were according to Silva et al. (2020): temperature 37 °C; 60 frames/s; minimum contrast, 30; straightness threshold, 80%; low-velocity average pathway (VAP) cutoff, 30.0 μm/s; and programming minimum VAP, 70.0 μm/s; straight line velocity (VSL) cutoff, 20.0 μm/s; and cell size, 8 pixels.

### In vitro maturation of domestic cat oocytes

Ovaries were recovered from healthy adult (1–2 years old) domestic cats after ovariectomy and transported to the laboratory in saline (NaCl 0.9%; 35–37 ºC). The oocytes were recovered by ovarian slicing and selected for the experiment. For each animal a different lot of oocytes (4 lots, 4–6 ovaries/lot) was used. Oocytes with one or more layers of *cumulus* cells and homogeneous cytoplasm were subjected to *in vitro* maturation (IVM) in a medium composed of TCM-199 (Gibco-BRL, Carlsbad, USA) supplemented with 3 mg/mL BSA, 0.1 mg/mL cysteamine, 1.4 mg/mL HEPES, 0.25 mg/mL sodium pyruvate, 0.6 mg/mL sodium lactate, 0.15 mg/mL L-glutamine, 20 μg/mL FSH/LH (Pluset®, Hertape Calier, Barcelona, Spain), and 1% antibiotic–antimycotic solution (Jewgenow et al., 2019). Approximately 10–15 oocytes were incubated in drops of 100 µL of the medium at 38.5 °C and 5% CO_2_ for 24 h.

### Heterologous in vitro fertilization and embryonic development

After IVM, the oocytes were washed twice in an IVF medium composed of TALP supplemented with heparin (10 µg/mL) (Veraguas et al., 2020). Oocytes (10–15 per drop) were co-incubated with 2 × 10^6^ sperm/mL in 50 µL drops of IVF medium for 18 h at 38.5 °C and 5% CO_2_. Next, the presumptive zygotes were washed, denuded slowly by pipetting, and incubated in 50 µL drops of the medium covered with mineral oil. The medium used for *in vitro* embryo development (IVD) consisted of synthetic oviductal fluid (SOF) supplemented with 0.2 mM sodium pyruvate, 0.2 mM L-glutamine, 0.34 mM sodium citrate, 2.8 mM myo-inositol, 2% essential amino acid solution, 1% non-essential amino acid solution, 1% antibiotic–antimycotic solution, 5 mg/mL BSA, and 2.5% fetal bovine serum (FBS, Gibco-BRL, Carlsbad, USA). The day of fertilization was considered as day 0, and culture was carried out until day 7. On day 5, 50% of the medium was replaced with fresh medium.

### Evaluation of fertilization and embryonic development

The stages of embryonic development were evaluated by observing the morphology of the embryos. On day 2 of IVD, the total cleavage rate and number of embryos containing 2 cells and more than 3 cells were quantified. On day 5, the rate of morula formation was evaluated for embryos having more than 16 cells. On day 7, the number of blastocysts was quantified.

Moreover, after 48 h, non-cleaved oocytes were stained to assess their maturation and fertilization status. The oocytes were fixed in 4% paraformaldehyde diluted in phosphate buffer solution (PBS) for 30 min, washed, and stained with 10 µg/mL Hoechst 33342 for 15 min. Meanwhile, the presumptive zygotes were transferred to slides and visualized by fluorescence microscopy. Oocytes with first polar bodies and metaphase plates were matured. Oocytes were considered fertilized when sperm had penetrated the ooplasm and the second polar body as well as two pronuclei were visible.

### Scanning electron microscopy (SEM)

Embryos were processed for SEM following the protocol described by Vanroose et al. (2000) with some alterations. Briefly, the embryos were fixed for 2 h in 2.5% glutaraldehyde and 0.1 M cacodylate buffer, pH 7.2, at 4 °C. They were then washed twice for 5 min in 2% cacodylate buffer and post-fixed in 1% osmium tetroxide for 2 h at 25 °C. The embryos were washed again in distilled water for 10 min, dehydrated with increasing concentrations of ethanol, and mounted on glass coverslips. After drying, the embryos were coated with gold. Finally, ultrastructure of the embryonic cells was visualized using a scanning electron microscope (TESCAN VEGA3; Tescan Analytics, Fuveau, Bouches-du-Rhône, France).

### Statistical analyses

Each experiment was performed using sperm samples frozen in ACP-117c and Tris from four animals, totaling four replicates per extender. All data are expressed as the mean ± standard error and were analyzed using the StatView 5.0 (SAS Institute Inc., Cary, NC, USA). Normality of all results were verified with the Shapiro–Wilk test and homoscedasticity was verified with the Levene’s test. The Fisher exact test was applied to evaluate the effect of each extender on the analyzed variables. Statistical significance was set at *p <* 0.05.

## Results

Immediately after semen collection, the average sperm motility evaluated subjectively was 95 ± 0% and concentration was 137.5 ± 31.2 × 10^6^ sperm/mL. An average of 72.5% normal sperm was found. After thawing and centrifugation, the total motility assessed using CASA was 9.0 ± 5.2% for sperm frozen in ACP-117c and 21.5 ± 6.3% for Tris. The progressive motility was of 0.3 ± 0.3% for ACP-117c and 7.5 ± 5.9% for Tris. The results of each animal are shown in Table 1.

**Table 1:** Individual results of animals regarding collection and motility after thawing.

**Animal**	**Subjective motility (%)**	**Volume (mL)**	**Concentration (× 10^6^)**	**Normal morphology (%)**	**ACP-117c (%)**		**Tris (%)**	
					**TM**	**PM**	**TM**	**PM**
1	95.0	7.5	120.0	74.5	24.0	1.0	21.0	1.0
2	95.0	6.3	220.0	76.5	1.0	0.0	31.0	25.0
3	95.0	6.0	140.0	79.0	8.0	0.0	30.0	4.0
4	95.0	5.5	70.0	60.0	3.0	0.0	4.0	0.0
Mean ± standard error	95.0 ± 0.0	6.3 ± 0.4	137.5 ± 31.2	72.5 ± 4.3	9.0 ± 5.2	0.3 ± 0.3	21.5 ± 6.3	7.5 ± 5.9

TM: Total motility (CASA); PM: Progressive motility (CASA).

**Figure 1:**
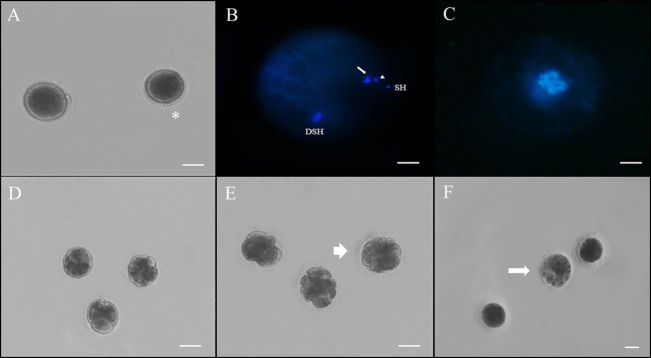
Representative images of retrospective assessment of maturation and fertilization and embryonic development. **(A)** Cat oocyte showing extrusion of the first and second polar body (indicated by the asterisk); **(B)** Fertilized oocyte with extrusion of the second polar body stained with Hoechst 33342 (DSH: decondensed sperm head; SH: sperm head attached to the zona pellucida; Arrow: extrusion of the second polar body; Arrowhead: first polar body); **(C)** Fertilized oocyte with pronucleus formation; **(D)** Cleaved embryos with more than three cells; **(E)** Embryo in early morula stage evaluated in D5 (arrow); **(F)** Low quality early blastocyst (arrow) assessed at D7. Scale bar: A–E: 100 µm; F: 200 µm.

Frozen-thawed sperm obtained from the jaguars were able to fertilize domestic cat oocytes (Table 2). Considering non-cleaved oocytes after 48 h of IVD, the total fertilization rate was in the range of 13.7 ± 2.8% to 17.9 ± 11.1% as observed by the presence of the second polar body (Figure 1A and B) and two pronuclei (Figure 1C). However, no significant difference was observed in fertilization parameters using sperm frozen in ACP-117c or Tris (Table 2).

**Table 2:** Retrospective evaluation of non-cleaved oocytes in relation to maturation and fertilization after 48 h of heterologous fertilization between cryopreserved jaguar sperm and cat oocytes.

**Extenders**	**Matured oocytes/total non-cleaved, %**	**Second polar body/total non-cleaved, %**	**Two pronucleus/total non-cleaved, %**	**Fertilized oocytes/total non-cleaved, %**	**Fertilized oocytes/ total non-cleaved matured, %**
ACP-117c	70.6 ± 12.7 (36/51)	3.9 ± 2.9 (2/51)	9.8 ± 4.7 (5/51)	13.7 ± 2.8 (7/51)	19.4 ± 5.0 (7/36)
Tris	66.1 ± 11.9 (37/56)	3.6 ± 3.1 (2/56)	14.3 ± 8.0 (8/56)	17.9 ± 11.1 (10/56)	27.0 ± 13.8 (10/37)
*P value*	*0.708*	*0.967*	*0.723*	*0.760*	*0.670*

Mean (4 animals) ± standard error (total number of oocytes). Degenerate oocytes were not included in the total non-cleaved oocytes.

During embryonic development, hybrid embryos of jaguar and cat were observed at the cleavage, morula, and blastocyst stages (Figure 1DF). The development rates of the embryos were similar between the two extenders and are shown in Table 3. On the second day of IVD, the cleavage rate varied from 16.7 ± 3.6% to 22.9 ± 3.2% between the groups, and embryos with 2 or more cells were found. Additionally, a total of seven morulae were obtained from sperm frozen in ACP-117c and Tris extenders. Moreover, only from samples frozen in Tris during the freezing of jaguar spermatozoa, it was possible to obtain two blastocysts that presented poor morphological quality (Figure 1F).

**Table 3:** Results of cleavage (D2), morula (D5) and blastocysts (D7) after heterologous fertilization between cryopreserved jaguar sperm and cat oocytes.

**Extenders**	**Cleavage, %**			**Morula/ cultured oocytes, %**	**Blastocyst/cultured oocytes, %**	**Blastocyst/total cleaved, %**
	**Total cleaved/cultured oocytes**	**2 cell/total cleaved**	**>3 cell/total cleaved**			
ACP-117c	22.9 ± 3.2 (16/70)	43.8 ± 7.4 (7/16)	56.3 ± 7.4 (9/16)	4.3 ± 2.3 (3/70)	0.0 ± 0.0 (0/70)	0.0 ± 0.0 (0/16)
Tris	16.7 ± 3.6 (12/72)	58.3 ± 18.9 (7/12)	41.7 ± 18.9 (5/12)	5.6 ± 2.2 (4/72)	2.8 ± 1.5 (2/72)	16.7 ± 1.5 (2/12)
*P value*	*0.237*	*0.704*	*0.703*	*0.705*	*0.134*	*0.154*

Mean (four animals) ± standard error (total number of oocytes).

**Figure 2:**
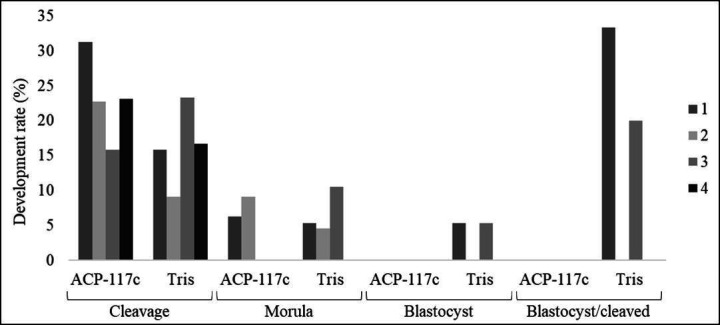
Development of embryos in different stages (cleavage, morula, and blastocyst) of four male jaguars from heterologous IVF with domestic cat oocytes.

Fertility of sperm per animal (Figure 2) was 50% according to the number of blastocysts obtained from the group of frozen sperm in Tris. According to the number of morulae obtained, the fertility of the animals was 50% with ACP-117c (2/4) and 75% (3/4) using Tris. Nevertheless, cleaved embryos were obtained from all the animals (100%, 4/4), independent of the extenders used.

**Figure 3:**
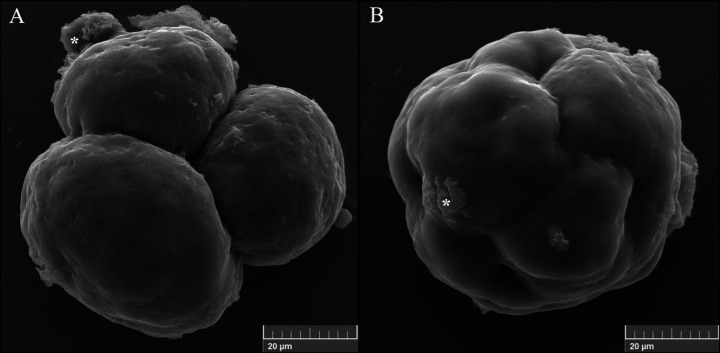
Ultrastructure of hybrid jaguar-cat embryos visualized by scanning electron microscopy. **(A)** Cleaved embryo with smooth surfaces and blastomeres with regular sizes; **(B)** Morula with smooth surfaces, compacted blastomeres flattening against one another, and the absence of cracks between embryonic cells. Asterisk indicates remaining fragments of the pellucid zone.

Due to processing for ultrastructural evaluation with SEM, the embryonic zona pellucida was lost, allowing visualization of embryonic cells and their surfaces (Figure 3). It was possible to observe embryonic cells with smooth surfaces and regular sizes as well as residual fragments of the pellucid zone. Figure 3A shows a hybrid jaguar and cat embryo with relatively spherical blastomeres in close contact, but without compaction. As shown in Figure 3B, morula with compacted blastomeres flattened against one another and absence of cracks among embryonic cells was observed.

## Discussion

To the best of our knowledge, the present work is the first report of *in vitro* development of hybrid embryos produced from frozen-thawed jaguar sperm and domestic cat oocytes. In general, cryopreservation caused severe damage to sperm motility, with a drastic drop-in motility rate, from 95.0% to 9.0% (ACP-117c) and 21.5% (Tris). This indicates that the cryopreservation protocol should be improved for jaguar sperm.

Only using Tris was it possible to obtain embryos in the blastocyst stage; however, there was no statistical difference between the extenders, probably due to the number of samples used. Even so, the blastocyst rate achieved in the Tris group is encouraging for further studies in semen cryopreservation and embryo production in jaguars. This result may be related to better quality of parameters of jaguar semen preserved in Tris, as previously demonstrated by Silva et al. (2020). These authors observed better motility (46.0% *vs*. 20.9%), membrane functionality (60.5% *vs*. 47.1%), and mitochondrial activity (21.5% *vs*. 11.8%) in samples cryopreserved in Tris rather than those cryopreserved in ACP-117c. Similarly, Tris promoted better preservation of the quality of frozen-thawed sperm from domestic cats compared to that by ACP-117c (Barbosa et al., 2020).

The positive effect of Tris can be attributed to its buffering property, which shows resistance to pH changes that occur during cooling for cryopreservation (Silva et al., 2011). Moreover, this extender has osmotic activity, low toxicity, and antioxidant action due to the presence of citric acid in its composition (Niasari-Naslaji et al., 2006). Another interesting feature of Tris is that less fructose is consumed by the sperm from this medium; this makes the energetic substrate available for a longer period for sperm survival (Miyasaki et al., 2014; Silva et al., 2020).

The low fertilization rate can be attributed to poor sperm quality after thawing. Despite the low percentage of motility post-thaw and centrifugation, jaguar sperm fertilized 19 to 27% of the matured oocytes of cats. This is probably because IVF performed in small drops (50 μL) facilitates an easy encounter of mobile sperm with oocytes. Our results are similar to those previously observed for the clouded leopard (*Neofelis nebula*), in which the fertilization rate of cat oocytes ranged from 15% to 29.4% using samples having motility in the range of 27.1% to 32.1% (Tipkantha et al., 2016). Moreover, in the bobcat, this rate reached 46% with a sperm motility index (sperm% motility + (progressive motility × 20)/2) of approximately 50% (Gañán et al., 2009).

The differences observed in results among felids may be due to the needs of each species regarding conditions of manipulation of sperm. Thuwanut et al. (2011) used a similar protocol for heterologous IVF of flat-headed cat sperm with domestic cat oocytes and for homologous IVF for domestic cats. The authors observed a better development of embryos in homologous cat IVF (blastocyst rate: 13.6% *vs*. 4%), showing that the main problem was related to sperm quality and not the IVF system. In a study carried out in puma (*Puma concolor*), it was believed that the sperm were of high quality because the rate of cleavage (49.0%), morula (34.4%), and blastocyst (23.4%) formation was similar to that of homologous embryos of domestic cats (67.1%, 47.8%, and 30.1%, respectively) (Duque et al., 2018).

Jaguar sperm were able fertilize cat oocytes, and the few resulting embryos developed to the blastocyst stage. The rate of production of hybrid jaguar-cat embryos was low, however, it was in line with the results obtained from other felids, such as bobcat (*Lynx rufus*), which had a cleavage rate of 17.2% to 26.5% using frozen sperm with TES-Tris (Gañán et al., 2009) and flat-headed cats that had a blastocyst rate ranging from 1 to 4% using frozen sperm with Tris (Thuwanut et al., 2011). In the present study, we did not perform parthenogenetic activation in cat oocytes, but it was observed that the rate of spontaneous embryo formation after IVF with clouded leopard sperm ranges from 0.8% to 1.6% (Tipkantha et al., 2016). Moreover, cat oocytes that undergo parthenogenetic activation after intracytoplasmic injection do not normally develop beyond the cleavage stage (Buarpung et al., 2013; Moro et al., 2014).

It is difficult to have females available for artificial insemination and oocyte harvesting; hence, our results are relevant because they show that oocytes from cats can be used efficiently to evaluate the *in vitro* fertility of sperm from wild felids. This is probably because some fertilization mechanisms are conserved among phylogenetically close species, allowing the development of hybrid embryos (Bedford-Guaus et al., 2011). Nevertheless, it is necessary to improve the protocols for processing and cryopreserving the semen of these animals.

The morphological quality of the hybrid jaguar-cat blastocysts was poor. Moreover, using SEM, it was observed that the surface of the embryo blastomeres was smooth, which was different than that observed in other species, whose blastomeres surfaces were covered by microvilli in bovine, rabbit (Koyama et al., 1994), and mouse (Li et al., 2011). These microvilli are related to the process of compacting and polarizing the blastomeres, which is responsible for differentiating the internal cell mass and the trophectoderm (Li et al., 2011). The absence of these microvilli may explain the low quality of the jaguar-cat blastocysts, in which the internal cell mass was heterogeneous.

## Conclusion

In conclusion, frozen-thawed jaguar sperm cryopreserved in ACP-117c or Tris extenders can fertilize and activate domestic cat oocytes for embryonic development. Using Tris, but not ACP-117c, it was possible for hybrid embryos to develop to the blastocyst stage, which, despite the low quality, are strong indications of sperm *in vitro* fertility. These results are fundamental for the development of a jaguar semen bank, which can contribute to conservation of the species by the application of assisted reproduction techniques *in vivo* and *in vitro*.
